# Lipoteichoic acid is an important microbe-associated molecular pattern of *Lactobacillus rhamnosus* GG

**DOI:** 10.1186/1475-2859-11-161

**Published:** 2012-12-15

**Authors:** Ingmar JJ Claes, Marijke E Segers, Tine LA Verhoeven, Michiel Dusselier, Bert F Sels, Sigrid CJ De Keersmaecker, Jos Vanderleyden, Sarah Lebeer

**Affiliations:** 1 Centre of Microbial and Plant Genetics, K U Leuven, Kasteelpark Arenberg 20, B-3001, Leuven, Belgium; 2 Department of Bioscience Engineering, University of Antwerp, Groenenborgerlaan 171, B-2020, Antwerp, Belgium; 3 Center for Surface Chemistry and Catalysis, KU Leuven, Kasteelpark Arenberg 23, 3001, Leuven, Belgium

**Keywords:** Lipoteichoic acid, Probiotics, Immunomodulation, Pro-inflammatory

## Abstract

**Background:**

Probiotic bacteria are increasingly used as immunomodulatory agents. Yet detailed molecular knowledge on the immunomodulatory molecules of these bacteria is lagging behind. Lipoteichoic acid (LTA) is considered a major microbe-associated molecular pattern (MAMP) of Gram-positive bacteria. However, many details and quantitative data on its immune signalling capacity are still unknown, especially in beneficial bacteria. Recently, we have demonstrated that a *dltD* mutant of the model probiotic *Lactobacillus rhamnosus* GG (LGG), having modified LTA molecules, has an enhanced probiotic efficacy in a DSS-induced colitis model as compared to wild-type.

**Results:**

In this study, the importance of D-alanylated and acylated LTA for the pro-inflammatory activity of LGG was studied *in vitro*. Purified native LTA of LGG wild-type exhibited a concentration-dependent activation of NF-κB signalling in HEK293T cells after interaction with TLR2/6, but not with TLR2 alone. Chemical deacylation of LTA interfered with the TLR2/6 interaction, while a moderate effect was observed with chemical dealanylation. Similarly, the *dltD* mutant of LGG exhibited a significantly reduced capacity to activate TLR2/6-dependent NF-κB signalling in a HEK293T reporter cell line compared to wild-type. In addition, the *dltD* mutant of LGG showed a reduced induction of mRNA of the chemokine IL-8 in the Caco-2 epithelial cell line compared to wild-type. Experiments with highly purified LTA of LGG confirmed that LTA is a crucial factor for IL-8 mRNA induction in Caco-2 epithelial cells. Chemical dealanylation and deacylation reduced IL-8 mRNA expression.

**Conclusions:**

Taken together, our results indicate that LTA of LGG is a crucial MAMP with pro-inflammatory activities such as IL-8 induction in intestinal epithelial cells and NF-κB induction in HEK293T cells via TLR2/6 interaction. The lipid chains of LGG LTA are needed for these activities, while also the D-alanine substituents are important, especially for IL-8 induction in Caco-2 cells.

## Background

The immunomodulatory potential of probiotic bacteria is of increasing interest for prophylactic and therapeutic options in various complex disorders, ranging from diarrhea to allergy. However, clinical and intervention studies with probiotics are not universally effective. For instance, the well-known probiotic *Lactobacillus rhamnosus* GG (LGG) shows promising results in the prevention of atopic disease [1-3], prevention and relief of diarrhea [4] and prevention of respiratory tract infections in children [5], while the results in Crohn’s disease are less promising [6-8]. Clearly, the mode and timing of delivery and severity of the conditions are important factors in determining probiotic efficacy. To select the optimal strain for each application, detailed molecular knowledge would be of great help. Many immunomodulatory molecules of bacteria are microbe-associated molecular patterns or MAMPs that can interact with pattern recognition receptors (PRRs) such as Toll-like receptors (TLRs). PRRs expressed on host cells such as intestinal epithelial cells (IECs) and dendritic cells (DCs) in the gastro-intestinal tract appear to be especially crucial for probiotic-host interactions. The interaction between a MAMP and a PRR on host cells results in the induction of signalling cascades that mount a molecular response. This response can include the production of pro- or anti-inflammatory cytokines and chemokines, antimicrobial molecules and cytoprotective factors [9].

Many MAMPs are surface located molecules, such as lipopolysaccharides interacting with TLR4/MD2 and flagellin interacting with TLR5. Lipoteichoic acid (LTA) is a widely studied MAMP of the low G+C subdivision of the Gram-positive bacteria such as the *Firmicutes*. LTA is an amphiphilic molecule of which the hydrophilic region is generally composed of a 1,3-phosphodiester-linked polymer of glycerol-phosphate or ribitol-phosphate variously substituted with D-alanine or sugars. The hydrophobic region is a glycolipid, often Glc(β1-6)Glc(β1-3)diacylglycerol [10]. LTA of several pathogens such as *Staphylococcus aureus*, *Streptococcus pyogenes* and *Streptococcus pneumoniae* has been shown to trigger the production of pro-inflammatory cytokines, nitric oxide (NO) and other pro-inflammatory mediators in various cell types [10,11]. Similar to LPS, LTA appears to be an important endotoxin capable of causing sepsis. However, whereas LPS is active in the nanomolar range, biological activities of LTA are mostly observed in the micromolar range [11]. Moreover, the activity of LTA is often debated due to problems with contaminants such as lipoproteins and loss of bio-active residues during isolation and purification [12]. Some studies have indicated that TLR2 is an important PRR for pathogenic LTA of *Streptococcus pneumoniae*[13] and *Staphylococcus aureus*[14], but generally without detailed mechanistic insights.

Recently, LTA molecules of probiotic lactobacilli have gained interest as important immune modulators based on studies with LTA mutants showing enhanced efficacy in experimental colitis models [15]. We observed previously that LGG wild-type can aggravate colitic symptoms in a DSS-induced colitis model in mice, suggesting that pro-inflammatory molecules of LGG itself can trigger an enhanced inflammatory response in certain conditions such as in severely progressed colitis [16]. In contrast to LGG wild-type, adding the *dltD* mutant of LGG seemed to improve colitic symptoms compared to the PBS control. This *dltD* mutant was previously shown to lack D-Ala on its LTA, but also expresses a modified glycolipid anchor and a shorter polyglycerol-P chain [17]. Similarly, a *dlt* mutant with modified D-alanylation of LTA was shown to have a higher anti-inflammatory potential in *L. plantarum* NCIMB8826 [18], while LTA removal in *L. acidophilus* NCFM conferred improved anti-inflammatory properties in several mouse models for IBD [19]. However, only very limited data exist on the structure-activity relationship of LTA of probiotic lactobacilli. In this study, we aimed to provide molecular insights in the biological activity of LTA of the widely used and model probiotic strain LGG, by investigating its potential pro-inflammatory activity and its interaction with PRRs in cell models.

## Results

### Isolation and chemical characterization of native and modified LTA of LGG

**Figure 1:**
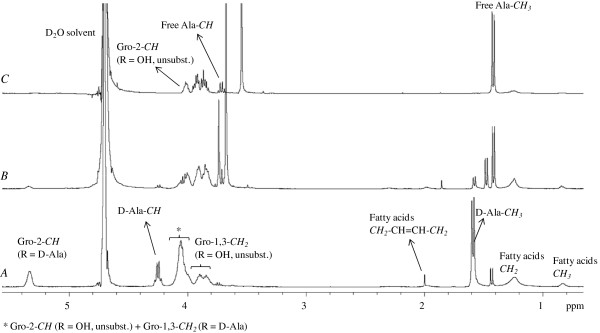
**NMR spectra of native and chemically modified lipoteichoic acid (LTA) of LGG. A**) ^1^H-NMR spectrum (600 MHz) of a representative sample of purified LTA from LGG wild type. **B**) ^1^H-NMR spectrum of purified LTA chemically treated to mainly remove D-alanine esters by alkaline hydrolysis in Tris buffer in D_2_O pH 8.5 for 24 h at 25°C. **C**) ^1^H-NMR spectrum of purified LTA treated by alkaline hydrolysis in 0.1M NaOH for 2 h at 37°C for chemical deacylation. Signals at 3.67 ppm (in B) and 3.56 ppm (in C) are due to the Tris buffer.

Highly purified LTA of LGG was isolated as described in Methods. In order to verify its structure, purified LTA was analysed with ^1^H-NMR. Hereto, 5 mg of purified LTA was dissolved in D_2_O. The obtained spectra were compared to ^1^H-NMR-spectra described in literature and showed to have > 99% purity (Figure 1).

**Figure 2:**
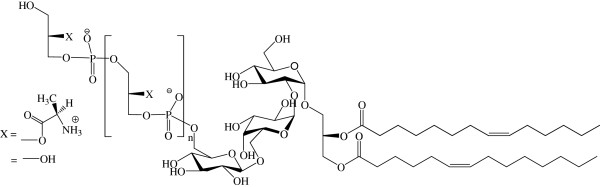
**Schematic representation of the structure of native LTA from LGG wild-type determined by NMR spectroscopy analysis.**^1^H-NMR spectra were analysed and integrated with Topspin 2.0 software. Based on the integrations of the corresponding signals in the spectra, the chain length (n) of the polyglycerolphosphate backbone was estimated between 30 and 78 depending on a biological variability (4 samples were tested). The second carbon of every glycerol was substituted with X, being either a hydroxyl group or D-alanine. The two acyl chains of LTA each have one double bound (of which the exact position was not determined) and have a total length of approximately n=14 (based on [17] and confirmed by integration data from this study). For the biological LTA sample of LGG wild-type (see also Figure 1), N = 30, while X = 71.8% D-Ala. For mLTA1, X = 15.6% D-Ala, while X = 0% for mLTA2. For mLTA2, 55% of the acyl chains of LTA were found to be removed.

The molecular structure determined by ^1^H-NMR spectroscopy confirmed that isolated LTA from LGG wild-type cells consists of a glycerolphosphate backbone decorated with D-alanyl esters as unique detectable substituents. The D-alanine content was calculated based on the integral ratios to be 71.8% (see structural representation in Figure 2 based on NMR spectra in Figure 1A). The chain length (n) of the glycerophosphate residues in LTA from LGG wild-type in the LTA sample analysed was around 30, based on the ^1^H NMR integrals of native LTA (Figure 1A). Some variation was noted between different biological samples with values ranging between n = 30 and n = 76 (a representative LTA sample is shown in Figure 1). Analysis of the glycolipid moiety reveals an average fatty acid chain of C14, with one double bond per fatty acid (two double bonds in every membrane anchor). The position of this double bond cannot be verified from these spectra and is therefore arbitrarily represented in Figure 2. LTA was also isolated from the *dltD* mutant of LGG, but NMR analysis showed too much impurities for detailed studies. As an alternative, purified LTA from LGG wild-type was chemically deacylated and dealanylated for determination of the structure-activity relationships by two different treatments (moderate and harsh alkaline hydrolysis) based on [20] and structural changes were confirmed by NMR. A moderate alkaline hydrolysis in Tris buffer pH 8.5 for 24 h at 25°C resulted in a drastic reduction of D-Ala ester substitutions. More specifically, a 71.8% to 15.6% reduction in the degree of substitution was noticed, based on the integration ratios of chemical shifts (δ)_H_ 5.35 ppm, 4.25 ppm and 1.65 ppm (see Figure 1B). Only a minor effect on the acyl chains of the LTA was observed. (Compare signals at (δ)_H_ 1.2 ppm - CH_2_ (γ-ω) of the fatty acid chains - with those at (δ)_H_ 0.8 ppm - the terminal CH_3_ - in Figures 1A and 1B). The harsh alkaline hydrolysis treatment with 0.1M NaOH for 2 h at 37°C significantly reduced the acyl chains (from 100% to 45%) and removed all D-Ala substitutions (Figure 1C). In the remaining part of the manuscript, these chemically modified LTAs will be termed as mLTA1 (isolated LTA treated by mild alkaline hydrolysis, which results in partial removal of D-Ala) and mLTA2 (isolated LTA treated by harsh alkaline hydrolysis, which completely removed D-Ala residues and resulted in partial acyl chain removal) respectively.

### NF-κB activation of LGG LTA by interaction with TLR2/6

**Figure 3:**
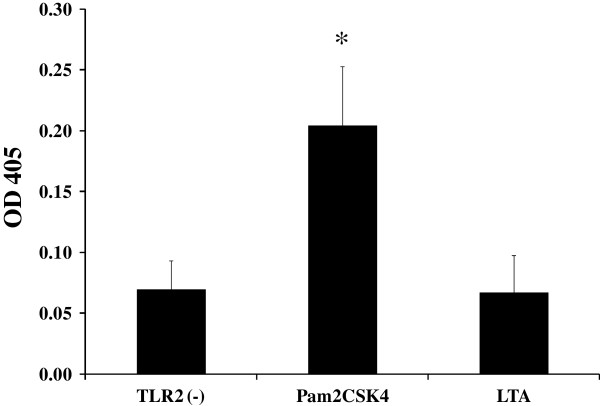
**TLR2 mediated signalling using HEK293T cells.** HEK293T cells were transfected with pUNO-hTLR2 (Invivogen) alone (TLR2 (−)) or together with the reporter plasmid pNiFty2-SEAP (Invivogen). Cells were transiently transfected with the corresponding plasmids as described in Material & Methods. 24h after transfection, cells were incubated with Pam2CSK4 (50 ng/ml) and LTA (10 μg/ml) for 24 hours. After incubation, NF-κB-induced SEAP activity was assessed using pNPP (p-nitrophynyl phosphate) and measuring the OD at 405nm. Data represent mean values ± SD. ^*^p-value < 0.05 compared to the negative control (TLR2(−)).

Since LTA of several bacteria, such as *Staphylococcus aureus* and *L. plantarum*, has been demonstrated to be a ligand for TLR2 [14,18], we first aimed to investigate the importance of this PRR for the signalling capacity of LTA of LGG. Hereto, we used HEK293T cells transiently expressing TLR2 and containing a NF-κB reporter plasmid. Surprisingly, expression of only TLR2 on the surface of the HEK293T cells was insufficient to mediate NF-κB activation by LTA of LGG (Figure 3).

**Figure 4:**
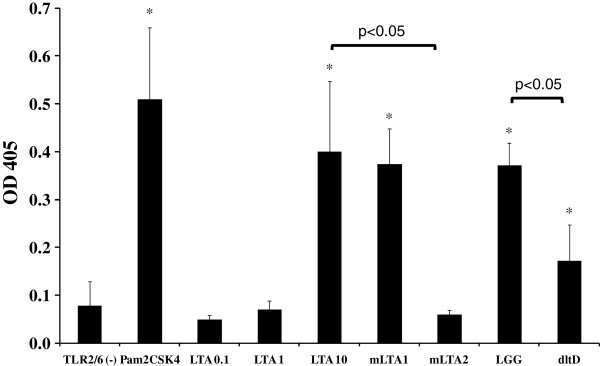
**TLR2/6 mediated signalling using HEK293T cells.** HEK293T cells were transfected with pDUO-hTLR6/TLR2 (Invivogen) alone (TLR2/6 (−)) or together with the reporter plasmid pNiFty2-SEAP (Invivogen). Cells were transiently transfected with the respective plasmids as described in Methods. 24h after transfection, cells were incubated with Pam2CSK4 (50 ng/ml), LTA at 0.1, 1 and 10 μg/ml, mLTA1 (10 μg/ml), mLTA2 (10 μg/ml), LGG wild-type or *dltD* mutant cells (10^8^ CFU/ml) for 24 hours. After incubation, NF-κB-induced SEAP activity was assessed using pNPP (p-nitrophynyl phosphate) and measuring the OD at 405nm. Data represent mean values ± SD. ^*^p-value < 0.05 compared to the negative control (TLR2/6(−)).

In contrast, transfecting the HEK293T cells with the vector pDUO-hTLR6/TLR2 (Invivogen), resulting in expression of both TLR2 and TLR6, did significantly result in activation of the NF-κB reporter plasmid by 10 μg/ml LTA of LGG (Figure 4). Lower concentrations of 0.1 and 1 μg/ml did not induce secreted alkaline phosphatase (SEAP) above baseline levels, which is in line with the literature [21].

### Importance of D-Ala substituents and acyl chains for TLR2/6 interaction by LGG LTA

To establish a structure-activity relation of LTA of LGG in interaction with TLR2/6 as PRRs, experiments were performed with chemically deacylated and dealanylated LTA, i.e. mLTA1 and mLTA2 described above. Partially modified mLTA1 showed a similar signalling through TLR2/TLR6 as native LTA, while the more severely deacetylated and fully dealanylated mLTA2 proved ineffective in activating NF-κB (Figure 4).

Interestingly, a similar trend was observed when investigating the interaction capacity of whole UV-irradiated bacterial cells. While LGG wild-type was able to significantly activate NF-κB signalling via TLR2/6 interaction, the *dltD* mutant, having modified LTA [17], significantly induced less NF-κB (Figure 4).

### IL-8 mRNA induction in intestinal epithelial cells by LGG LTA

**Figure 5:**
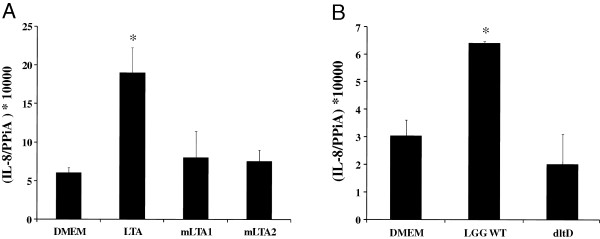
**Interleukin-8 mRNA response of human Caco-2 cells to 1.5 h stimulation with various compounds.** Cells were stimulated with LGG LTA, mLTA1 and mLTA2 (10 μg/ml) (panel **A**) or with live LGG WT or the *dltD* mutant strain (panel **B**) for 1.5 hours. DMEM medium served as a negative control. Bacteria were administered at 10^7^ CFUs per ml. The results shown are representative for at least three independent experiments. Data represent mean values ± SD. ^*^p-value < 0.05 compared to the negative control (DMEM).

Subsequently, we investigated the structure-activity relationship of LGG LTA in intestinal epithelial cells. Native LTA of LGG was previously shown to induce IL-8 mRNA in Caco-2 epithelial cells with a maximum activity around 10 μg/ml [22]. To investigate the role of the D-Ala substituents and the lipid part of LTA, mLTA1 and mLTA2 were used as described above. Both treatments resulted in a significant reduction in IL-8 mRNA induction, highlighting the importance of both the acyl groups and the D-Ala ester substituents of LTA for this activity (Figure 5A).

In addition, we investigated the IL-8 mRNA induction capacity of LGG wild-type versus the *dltD* mutant. Compared to the negative control (DMEM), LGG wild-type significantly induced IL-8 mRNA, while the *dltD* mutant did not (Figure 5B). Blocking experiments with anti-LTR2 antibodies confirmed the importance of TLR2 for IL-8 mRNA induction by LGG wild-type (data not shown).

## Discussion

In this study, we aimed to investigate the importance of the LTA structure of LGG in mediating pro-inflammatory effects in dedicated cell models. Hereto, we used highly purified native and chemically modified LTA of LGG. First, we showed that LGG LTA is able to activate NF-κB in HEK293T cells via interaction with the PRR couple TLR2/6. Importantly, this activity was not observed when the HEK293T cells were only transfected with TLR2, highlighting the importance of TLR6 as coreceptor for LGG LTA. This is in agreement with the PRRs of LTA molecules of *S. aureus* and group B streptococci, which were shown to interact with TLR2/6 and not TLR1/6 [23]*.* For lactobacilli, the pro-inflammatory potential of LTA of *L. plantarum* NCBIM8826 was shown to be TLR2 dependent using bone marrow cells isolated from TLR2^+/+^ and TLR2^−/−^ mice [18]. Experiments with LTA from *L. casei* and *L. fermentum* using HEK293T cells revealed that CD14 could only activate NF-κB when cotransfected with TLR2. Taken together, it seems that the pro-inflammatory character of LTA from different species is TLR2 dependent but needs coreceptors, such as TLR6 and/or CD14.

When we compared the activity of native with chemically modified LTA confirmed by NMR spectroscopy, i.e. mLTA1 (mild alkaline hydrolysis - partial removal of D-Ala) and mLTA2 (harsh alkaline hydrolysis - complete removal of D-Ala and partial acyl chain removal), D-alanylation does not appear to be crucial for TLR2/6 interaction. While a harsh chemical deacylation of 55% (mLTA2) completely abolished the NF-κB induction by LGG LTA, a severe chemical dealanylation of 72% (mLTA1) did not significantly affect this signalling. Our data confirm the essential role of the lipid part of LTA, as has been previously shown for other ligands in interaction with TLR2 [24]. Yet, they also suggest a modulatory role for the D-Ala substituents in signalling. This highlights the importance of structure-activity data for LTA of different species.

Subsequent experiments with the Caco-2 intestinal epithelial cells, a cell line that has been extensively used as a model for the intestinal barrier [25], indicate that LTA of LGG can indeed induce an inflammatory response, in this case a concentration-dependent increase in mRNA expression of the chemokine IL-8. Chemical dealanylation (mLTA1) was sufficient to significantly reduce IL-8 mRNA induction. These experiments suggest that the D-Ala substituents of LGG LTA are more important for IL-8 mRNA induction in Caco-2 cells than for NF-kB induction in HEK293T cells. This would be in agreement with the fact that full IL-8 mRNA induction in differentiated Caco-2 epithelial cells requires the interaction of LGG LTA with TLR2/6 and additional coreceptors interacting with the D-Ala substituents, which are not present in the less differentiated HEK293T cells. Furthermore, we could show in this study that the *dltD* mutant of LGG shows a significantly reduced pro-inflammatory capacity compared to wild-type, as assessed by TLR2/6-dependent NF-kB induction in HEK293T cells and IL-8 mRNA induction by Caco-2 cells. This is in agreement with its observed reduced pro-inflammatory and even enhanced anti-inflammatory and enhanced epithelial repair activity *in vivo*[16].

Due to pleiotropic effects of the *dltD* mutation, we were unable to isolate LTA from the *dltD* mutant that was sufficiently pure for immunomodulation experiments. Therefore, we are currently unable to answer the question as to whether the specific LTA structure of the *dltD* mutant as described in Perea Vélez *et al.*[17], results in this reduced pro-inflammatory capacity. Nevertheless, the data for NMR characterized chemical dealanylated and deacylated LGG LTA confirm the importance of both structural features for its pro-inflammatory potential.

In conclusion, we have convincingly shown in this study that LTA of LGG is an important MAMP of LGG for interaction with TLR2/6. Moreover, LTA is an important factor in the induction of pro-inflammatory markers in the Caco-2 intestinal epithelial cell line. This activity of LTA needs to be taken into account when selecting the optimal application for LGG. For example, in a disease situation like active colitis, extra pro-inflammatory stimulation by LTA of probiotic bacteria would be undesirable. Although human application of genetically modified probiotics such as the *dltD* mutant of LGG is not an option, experiments with mutants and isolated compounds such as shown in this study highlight the importance of tailored applications. Screening or searching for probiotic strains with less pro-inflammatory LTA seems to be important for specific disease conditions with a general pro-inflammatory profile. On the other hand, for application of probiotic strains or purified LTA as adjuvants, more immunostimulatory characteristics could be more appropriate.

## Methods

### Bacterial strains, media and growth conditions

LGG and the *dltD* mutant (CMPG5540) were grown at 37°C in MRS medium (Difco) or AOAC medium (Difco) under static conditions.

### LTA purification

Overnight LGG wild-type cell cultures were centrifuged for 30 min (4000 rpm, 4°C) and the white cell pellets were stored at −80°C. Highly purified LTA was isolated according to Morath *et al.*[26]. In order to release LTA from the peptidoglycan matrix, the cells were disrupted mechanically by sonication. Hence, a defrosted aliquot of bacteria (ca. 40 g) was suspended in 80 mL of citrate buffer (0.1 M, pH = 4). The milky solution was placed in an ice bath and 4 subsequent sonication steps were accomplished (duty cycle = 50%, output > 50). To remove the contaminant lipophilic cell molecules from the LTA containing buffer solution, an extraction step with n-butanone was applied as described by Morath *et al.*[20], as the classical extraction method with phenol-water at 68°C and subsequent dialysis was previously shown to partly desintegrate structure of the purified LTA.

The purification of LTA was accomplished by hydrophobic interaction chromatography (HIC) on an octyl-Sepharose CL-4B (GE Healthcare) packed in XK 16/60 column (GE Healthcare) using a linear gradient from 15% to 60% n-propanol in ammonium acetate (0.1 M, pH = 4.75). Hence, the lyophilized sample was suspended in 35 ml of chromatography start buffer (15% n-propanol in 0.1 M ammonium acetate, pH = 4.75) and centrifuged for 1 h (15000 rpm, 4°C). After sterilization by membrane filtration (0.2 μm), the supernatant was subjected to HIC.

The LTA containing fractions were identified by their phosphate content using the phosphomolybdenum test [27]. Therefore, to 50 μL of each fraction 200 μL of an acid solution (2 M H_2_SO_4_/0.44 M HClO_4_) was added and the samples were incubated at 140°C for 2 h. Next, a reduction solution (ammonium heptamolybdate solution: ascorbine solution 9:1) was added to each sample. After 2 h of incubation at 50°C, 200 μL of each sample was transferred into a microtiter plate. Absorption was measured at 700 nm. The phosphate content was determined by comparison to a P standard.

The LTA-containing fractions were collected and lyophilized after freezing with liquid N_2_. The lyophilized fractions were suspended in ca. 10 mL milli-Q water and lyophilized once more.

Dealanylation of LTA was achieved by increasing the pH of the water phase after butanol extraction, and by stirring at pH ≈ 8.5 with Tris buffer at RT (25°C) for 24 h as described previously [20]. Deacylation of LTA was performed at 37°C for 2 h with 0.1M NaOH [26]. LPS contamination was assessed with that LAL assay (<0.1 EU/ml; QCL-1000; Lonza).

### LTA structure analysis

Proton (^1^H) Nuclear magnetic resonance (NMR) spectra of the LTA samples were measured in 5 mm Norell tubes on a Bruker Avance II-plus 600 MHz spectrometer equipped with a TXI-5 probe at 298 K. Spectra were obtained using D_2_O as a solvent, with the residual solvent peak at 4.7 ppm. The data acquisition, post-processing and integrations were performed in Bruker Topspin 2.1 and 2.0. The length of the polyglycerophosphate backbone, the percentage of substitution of the second position of every glycerol with D-alanine, and the average chain length of the fatty acids in the membrane anchor were calculated using the appropriate integrated signals of the proton NMR spectra.

### Immunomodulation assay with Caco-2 cells

Caco-2 cells were purchased from ATCC (Rockville, MD). Cells were routinely grown in 75-cm^2^ culture flasks under conditions of 37°C, 5% CO_2_, and 90% relative humidity. Dulbecco modified Eagle medium (DMEM)/F-12 (GibcoBRL) (1:1) supplemented with 10% fetal bovine serum (FBS; HyClone) was used as the culture medium. Cells were passaged every 3 days (at 70 to 80% confluency) at a split ratio of 1 to 7. Epithelial cells were plated at a density of 10^4^ cells/cm^2^ in 12-well plates (Cellstar). Confluency was reached within 3 to 4 days after seeding, and monolayers were used for the experiments 5–7 days after seeding. Caco-2 cells were incubated with LGG wild type, the *dltD* mutant (10^7^ CFU/ml) or LTA (at different concentrations) for 1.5 hours.

### Analysis of cytokine mRNA levels by qRT-PCR

To determine cytokine mRNA levels by qRT-PCR, commercially available methodologies for performing total cellular RNA extraction (High pure RNA isolation kit, Roche), reverse transcription (SuperScript III first-strand Synthesis system, Invitrogen) and real-time DNA amplification (TaqMan Universal PCR Master Mix, Applied Biosystems) were applied. For real-time quantitative PCR (RT-qPCR) amplification, the StepOnePlus Real Time PCR system (Applied Biosystems, Lennik, Belgium) was used. All primers and probes were designed based on published sequences and chemically synthesized by Eurogentec (Seraing, Belgium). Purified plasmid DNA specific for each targeted cytokine gene served as cDNA plasmid standards and was used to quantify the respective cytokine in the test samples. The relative abundance of each mRNA species was measured by qPCR using 45 amplification cycles, with each cycle consisting of a denaturation step (94°C for 15 s) and a 1 min combined annealing-extension step (60°C). qPCR data are presented as a ratio of the mRNA level for cytokine genes over that of the housekeeping gene peptidylprolyl isomerase A (PPIA), one of the most stably expressed genes in Caco-2 cells [28].

### Transient transfection of HEK293T cells and measurement of alkaline phosphatase activity

HEK293T cells (ATCC, Manassas, VA, USA) were cultured in DMEM/F-12 (GibcoBRL) (1:1) supplemented with 6% fetal bovine serum (FBS; HyClone) and 50μg/mL gentamycin in 5% CO_2_, 37°C, RH 90%. The cells (0.5 x 10^6^ cells/mL) were seeded in 12-well plates. These cells were then transfected with either pNiFty-SEAP alone or in combination with pUNO1-hTLR2 or pDUO-hTLR6/TLR2 (Invivogen Inc., San Diego, USA) using branched polyethylenimine (MW ~ 25.000). Transfected cells were cultured for 24 h and then incubated with Pam2CSK4 (positive control from Invivogen), LTA, UV inactivated LGG or the *dltD* mutant for an additional 24 h. All experiments were performed at least two times. After incubation, NF-κB-induced secreted alkaline phosphatase (SEAP) activity was assessed using pNPP (p-nitrophynyl phosphate) at 1 mg/mL and measuring the OD at 405 nm.

### Statistics

Values are presented as mean ± SD. Difference were evaluated by the unpaired Student’s t test. A value of p ≤ 0.05 was considered to be significant.

## Competing interests

The authors declare that they have no competing interests.

## Authors’ contributions

IJJC and MES designed and performed part of the experiments, analysed the data and wrote the paper. TLAV performed the immunomodulation experiments with Caco-2 cells. MD performed the NMR analysis of the LTA. MD and BS participated in the analysis of the NMR data and editing of the paper. SCJDK, JV and SL participated in the design and coordination of the experiments, analysis of the data and writing of the manuscript. All authors read and approved the final manuscript.
